# The Incorporate Institute of Hygiene

**Published:** 1907-12-14

**Authors:** 


### THE INCORPORATED INSTITUTE OF HYGIENE.

On December 4 a highly successful and well-attended reception was held at the premises of the Incorporated Institute of Hygiene, in Devonshire Street, W. The guests were received by Sir W. H. Bennett, who has succeeded the late Sir William Broadbent as President of the Institute. A most interesting programme had been arranged, including demonstrations in bacteriology, preparation of vaccines, and the diagnosis of various specific diseases. In the Lecture Hall bioscopic views were on show, and an orchestra provided popular music. The rooms were full to overflowing, and the numerous visitors availed themselves freely of the samples which were lavishly distributed by enterprising firms of exhibitors.

It would be impossible to describe or even to mention all the exhibits in the different sections, which are devoted respectively to clothing, food, beverages, domestic hygiene, medical, surgical, and hospital appliances. When the system and *raison d'être* of the Institute are explained, it will be understood that its Devonshire Street Exhibition is well worth a visit from anyone interested in the subjects just mentioned. Briefly, the Institute exists for the purpose of improving the standard of domestic and personal hygiene, as distinct from the domain of purely State medicine. Its disinterested character and freedom from any suspicion of commercialism are guaranteed by the charter of Incorporation and by the names of the strong and representative Council. Any manufacturer desiring to bring before the medical profession, and, through it, before the public, wares the purity, ingenuity, convenience, or other advantage of which he wishes to emphasise, can exhibit samples of the same in the Institute on payment of certain charges: the Institute on its part undertakes to explain to visitors and inquirers the special points of each exhibit for which pre-eminence or excellence is claimed. But—and this is the kernel of the whole matter—before any such arrangement is concluded, the samples are submitted to analysis by a committee of experts, and the results of that analysis are compared with the advertised statements of the manufacturer concerning his goods. Moreover, this analysis is made not merely of the submitted samples, but also of the same articles bought through a retailer in the open market. If any distinct discrepancy is found between the manufacturer's claims and the disclosures of the analysis, an opportunity of affording an explanation is given; and unless this is satisfactorily forthcoming the exhibit is refused, at any rate until such time as the quality of the substance has improved. Similarly, articles the claims on behalf of which are found to be fraudulent, in respect either of purity, hygienic value, or failure to comply with the analysts' tests, are refused outright.

It will thus be seen how powerful a lever the Institute is able to exert in securing uniformity of standard and purity. All exhibitors receive yearly the certificate of the Institute in respect of their products and appliances, and it follows that a manufacturer who has anything to expect from the support of the medical profession cannot afford to risk a refusal of his samples. As long as the absolute *bona-fides* of such a corporation stand above suspicion, it is a court of appeal before whose decisions the defendant dare not fail to bow.

The exhibits are very numerous, and range from belts to beer, and from telephones to towels. Medicinal remedies are excluded entirely, as beyond the scope of the Institute, though of all such drugs as antiseptics, disinfectants, and so on, a large selection is on view. The appliance section is perhaps as interesting as any, and must be valuable in the extreme to anyone who is on the committee of a hospital, manages a nursing home, or, in short, is in any position of authority connected with arrangements for the reception of the sick. In this section the determination to mention no exhibit by name must be broken in favour of a most ingenious commode bed. The discomfort of every hitherto existing bed-slipper is one of which patients and medical men have long complained; in certain cases its use is both difficult and even dangerous. There need be no hesitation about saying that Mr. Heatley's invention, simple as it appears, will probably solve this difficult problem successfully. The test of time has not yet been applied to it, but it will be surprising if its reception into favour is not immediate.

It is not, of course, only in the appliance section that there are things worth seeing. In all the others there are novelties or improvements on existing goods which are coming to the front. The advantage of this permanent exhibition over the temporary medical exhibitions, sometimes held for commercial or advertisement purposes, is obvious: the mere existence of any product inside the walls of the Institute is a guarantee of its purity, and this is the keynote of the Incorporated Institute of Hygiene throughout.

